# Electrocorticography Evidence of Tactile Responses in Visual Cortices

**DOI:** 10.1007/s10548-020-00783-4

**Published:** 2020-07-13

**Authors:** Anna Gaglianese, Mariana P. Branco, Iris I. A. Groen, Noah C. Benson, Mariska J. Vansteensel, Micah M. Murray, Natalia Petridou, Nick F. Ramsey

**Affiliations:** 1 The Laboratory for Investigative Neurophysiology (The LINE), Department of Radiology, University Hospital Center, University of Lausanne, Rue Centrale 7, Lausanne, 1003 Switzerland; 2 Department of Neurosurgery and Neurology, UMC Utrecht Brain Center, University Medical Center Utrecht, Heidelberglaan 100, 3584 CX Utrecht, The Netherlands; 3 Department of Radiology, Center for Image Sciences, University Medical Center Utrecht, Heidelberglaan 100, 3584 CX Utrecht, The Netherlands; 4 Department of Psychology, New York University, Washington Place 6, New York, 10003 NY USA; 5 Sensory, Perceptual and Cognitive Neuroscience Section, Center for Biomedical Imaging (CIBM), Station 6, Lausanne, 1015 Switzerland; 6 Ophthalmology Service, Fondation Asile des aveugles and University of Lausanne, Avenue de France 15, Lausanne, 1004 Switzerland; 7 Department of Hearing and Speech Sciences, Vanderbilt University, 21st Avenue South 1215, Nashville, 37232 TN USA; 8 Present Address: eScience Institute, University of Washington, 15th Ave NE, Seattle, 98195 WA USA

**Keywords:** Multisensory, Tactile, High frequency band, ECoG

## Abstract

There is ongoing debate regarding the extent to which human cortices are specialized for processing a given sensory input versus a given type of information, independently of the sensory source. Many neuroimaging and electrophysiological studies have reported that primary and extrastriate visual cortices respond to tactile and auditory stimulation, in addition to visual inputs, suggesting these cortices are intrinsically multisensory. In particular for tactile responses, few studies have proven neuronal processes in visual cortex in humans. Here, we assessed tactile responses in both low-level and extrastriate visual cortices using electrocorticography recordings in a human participant. Specifically, we observed significant spectral power increases in the high frequency band (30–100 Hz) in response to tactile stimuli, reportedly associated with spiking neuronal activity, in both low-level visual cortex (i.e. V2) and in the anterior part of the lateral occipital–temporal cortex. These sites were both involved in processing tactile information and responsive to visual stimulation. More generally, the present results add to a mounting literature in support of task-sensitive and sensory-independent mechanisms underlying functions like spatial, motion, and self-processing in the brain and extending from higher-level as well as to low-level cortices.

## Introduction

Brain functional specialization has been historically described as the ability of single areas of the cortex to perform specific functions driven by different and distinct senses (e.g. the visual, auditory and somatosensory cortices). However, the concept of functional specialization has recently been challenged. For example, it has been shown in both sighted and blind individuals that the recruitment of some regions of the cortex is independent of the sensory modality in which the stimuli are presented (see for review: Amedi et al. 2017; Murray et al. 2016). The notion that visual areas are highly specialized to respond to only *visual* information has been challenged by a large number of studies demonstrating cross-modal convergence and multisensory integration with auditory or tactile responses in human visual cortices (Sadato et al. 1996; Zangaladze et al. 1999; Ghazanfar and Schroeder 2006; Lewis et al. 2010; Brang et al. 2019; Plass et al. 2019). In particular for tactile processing, pioneering studies using both PET and TMS suggested the recruitment of extrastriate visual areas close to the parieto-occipital sulcus in processing spatial information of tactile input (Sathian et al. 1997; Zangaladze et al. 1999). Activation of the Lateral Occipital Cortex LOC has been shown in response to haptic object recognition (reviewed in Amedi et al. 2017). Even primary visual cortex has been shown to be involved in Braille reading by the visually impaired (Sadato et al. 1996; Zangaladze et al. 1999). Moreover, in the last decades several neuroimaging studies have reported auditory and tactile responses to motion stimuli in the human Middle Temporal complex hMT+, more specifically in the most anterior part of the complex, known as visual extrastriate area MST (Blake et al. 2004; Van Boven et al. 2005; Beauchamp et al. 2007; Ricciardi et al. 2007; Ptito et al. 2009; Summers et al. 2009; Sani et al. 2010; Van Kemenade et al. 2014) and also in the human planum temporale (Battal et al. 2019). Other fMRI studies offer contradictory findings about the contribution of extrastriate cortex to tactile motion processing. Some have failed to observe significant activation in the hMT+ complex in response to tactile motion stimulation (Jiang et al. 2015). Though caution is necessary in the face of negative results, one reason for these controversial findings may reside in the imaging analysis performed by the different groups. Due to the nature of fMRI recordings, group average analysis is usually necessary, leading to potentially inaccurate localization of specific brain regions or blurring of localized responses.

In this context, intracranial recordings (electrocorticography, ECoG) provide a unique window to directly measure localized neuronal responses to different types of stimulation in humans. Given the high sensitivity and precise localization, ECoG recordings capture specific broadband spectral responses in the high frequency band (30–100 Hz) that have been linked directly to spiking neural activity (Miller et al. 2009; Ray and Maunsell 2011; Hermes et al. 2014). As such, ECoG provides both unprecedented temporal resolution and precise spatial localization in single participant space.

**Fig. 1:**
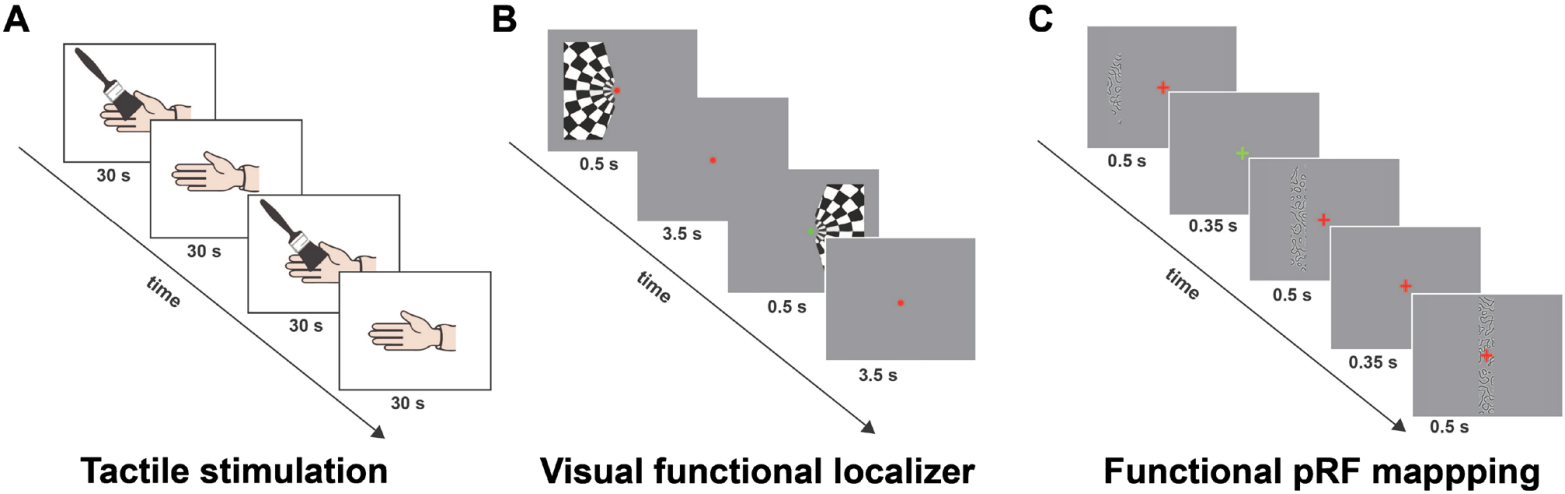
Experimental paradigms: **a** tactile stimulation, **b** visual functional localizer, **c** functional pRF Mapping

**Fig. 2:**
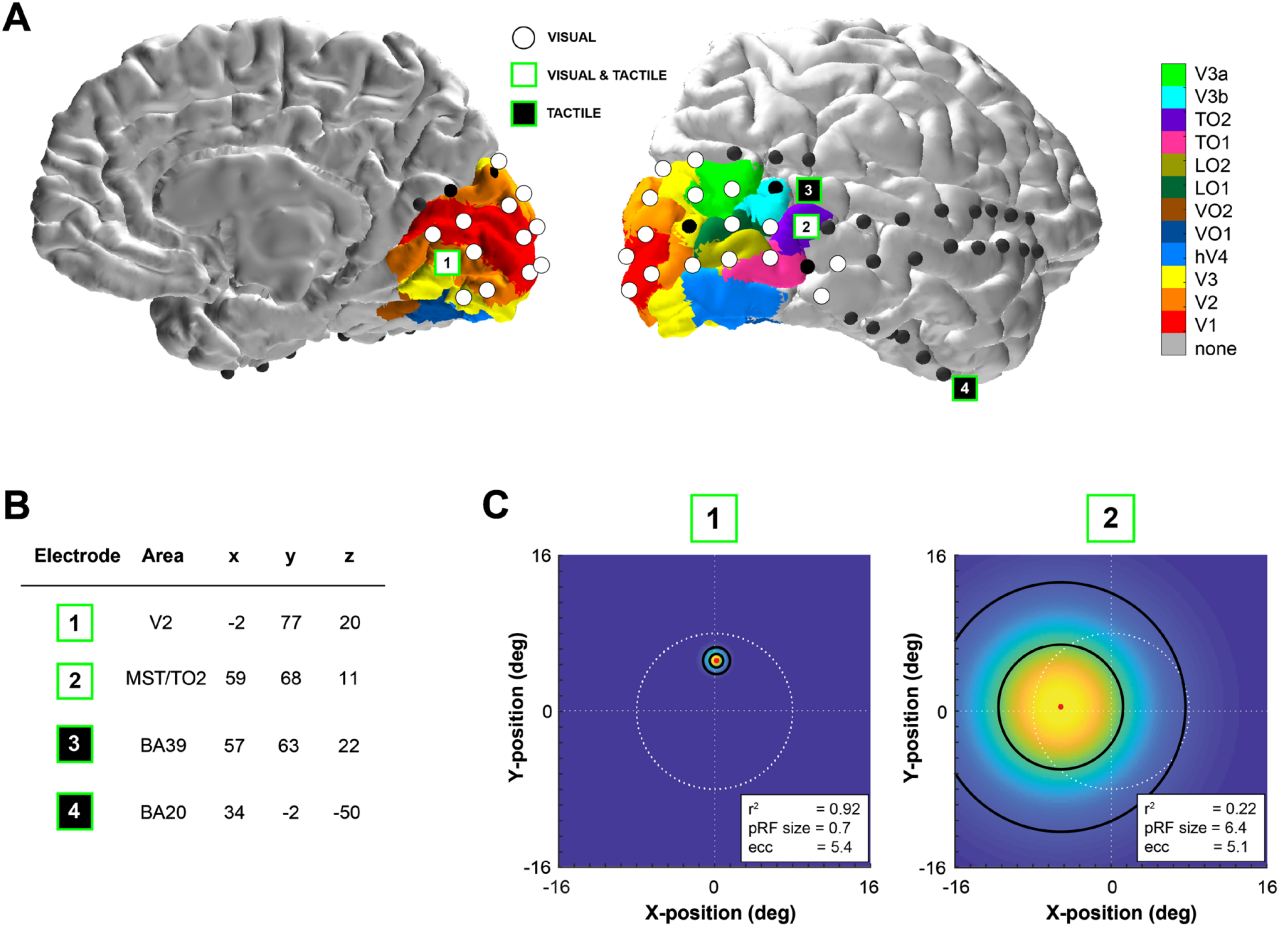
Spatial localization of significant high frequency band responses to tactile stimulation. **a** Electrodes exhibiting significant high frequency band responses to visual stimulation in either the contralateral or ipsilateral hemifield are shown in white on the participant’s brain rendering. Four electrodes (marked as 1 to 4) showed a significant change in high frequency band power during tactile stimulation. Electrodes responding to the tactile task and located in visually responsive sites are shown in white and green (electrode 1 and 2), while electrodes significantly responding to only the tactile task and not showing visual responses are displayed in black and green (electrodes 3 and 4). Colour maps indicate estimates of early and extrastriate visual areas based on the participant’s surface topology and a prior, learned retinotopic atlas (Benson et al. 2012; Benson and Winawer 2018). **b** Electrode coordinates in MNI space. **c** Estimated population receptive fields (pRFs) for electrodes 1 and 2 depicted in the visual field. White dashed lines indicate the central fixation (straight lines) and the extent of the visual field that was covered by the visual stimulus (16.6° diameter of visual angle, circle). The color scale indicates the height of the pRF, i.e. the best-fitting 2D isotropic Gaussian (yellow is high, blue is low), with the pRF center location indicated by the red dot, and black outlines depicting 1 and 2 pRF sizes. Corresponding pRF size and eccentricity parameters are presented in the lower right corner

Here, we recorded tactile and visual responses (Fig. 1) in a human participant implanted with intracranial electrodes covering primary and extrastriate visual cortices (Fig. 2). We observed high frequency band responses for both tactile and visual stimulation in low-level visual cortex (V2) and in the anterior part of the lateral occipital–temporal complex, which we contend is likely part of the hMT+ complex. In addition, significant responses to tactile (but not visual) stimulation were observed on the superior part of the middle temporal sulcus and the anterior ventral temporal lobe.

## Materials and Methods

The participant was a right-handed 20-year-old woman who underwent a subdural implantation of ECoG electrode grids as part of the clinical evaluation of her epilepsy. The participant was implanted with 64 electrodes (2.3 mm diameter surface and 1 cm inter-electrode spacing) covering most of dorsal medial and lateral visual cortex as well as temporal and ventral areas (Fig. 2). The medical ethical board of the Utrecht University Medical Center approved the study. The participant gave her written informed consent to participate in the study in compliance with the Declaration of Helsinki (2013). The participant performed: (i) a tactile stimulation, (ii) a functional localizer to define electrodes responsive to visual stimuli, and (iii) a visual population Receptive Field (pRF) mapping task to estimate visual electrodes’ receptive field properties (Dumoulin and Wandell 2008; Kay et al. 2019) (Fig. 1). The ECoG signals from all electrodes were acquired with a Micromed system at a sampling rate of 2048 Hz and high-pass and low-pass filters of 0.15 and 500 Hz, respectively. The patient also participated in ancillary scientific studies that included visual tests and passive movie watching.

### Tactile Stimulation

The participant laid in her hospital bed with her eyes closed and her right hand placed next to her, with her palm facing upward. The experimenter stood next to the bed and stroked back and forth along the palm of the participant’s right hand using a soft commercial brush during periods of 30 s (with a mean velocity of 1 stroke per second), alternated by 30-second periods of rest in a block design (Fig. 1). Visual instructions were presented on a computer screen to the experimenter only. Each block was repeated five times. The block design has been employed by neurologists in the Epilepsy Monitoring Unit (EMU) to obtain good quality data to functionally localize somatosensory areas in response to tactile stimulation in clinical settings.

### Functional Localizer of Visually-Responsive Electrodes

A visual functional localizer task was performed on a different day than the tactile stimulation. Stimuli of the visual functional localizer task were generated in Matlab and consisted of a unilateral dart-board pattern avoiding a central circular region (0.4° of visual angle) and displaced by 20° of polar angle from the vertical meridian. The stimulus radius was 10° of visual angle. The spokes of the dart-board pattern moved in opposite radial directions, each stimulus lasted for 0.5 s, and left (ipsi-lateral to the implanted electrodes) and right (contra-lateral) visual field stimulation were alternated with an interleaving 3 s baseline (grey screen), see Fig. 1. Both hemifields were stimulated to detect brain regions in visual cortex responding to visual stimulation in the entire visual field. The participant fixated on a dot located in the center of the screen and was instructed to press a button every time the dot changed from green to red colour. Stimuli were displayed on a 1024 × 768 pixel LCD screen of a Toshiba Tecra S10-101 laptop positioned at 75 cm distance from the participant’s eyes.

### ECoG Data Analysis

ECoG data were analysed using Matlab. Data quality for each electrode was evaluated by neurologists and one electrode showing artefacts located in the temporal lobe was removed from the analysis. For both the visual localizer and tactile stimulation, data were re-referenced to the common average of all remaining electrodes. Common average reference (CAR) has been proven to be effective in removing common noise across electrodes in ECoG datasets and to provide similar results as referencing to a silent electrode, which was located at the mastoid in this participant (Hermes et al. 2014; Liu et al. 2015; Biasiucci et al. 2019).

For the tactile stimulation, power spectral density was computed per electrode for tactile stimulation and baseline epochs, using Welch’s periodogram averaging method (1 Hz sampling and 1 s window). Tactile and baseline epochs were defined as the 30 s after the start of the brushing stimulation and the 30 s after tactile offset, respectively. A spectral elevation in the high frequency band (30–100 Hz) is consistently observed in task-related ECoG measurements and has been associated with neuronal spiking activity in response to sensory stimuli (Miller et al. 2009; Winawer et al. 2013; Hermes et al. 2014). Therefore, electrodes exhibiting significant responses for tactile stimulation were selected by statistically comparing the mean responses in the high frequency band (30–100 Hz) during active epochs to the mean power of the baseline epochs (paired t-test, P < 0.01 Bonferroni corrected for the total number of included electrodes). To additionally characterize the spectral power change during tactile stimulation in the complete time-frequency domain, we computed the spectrogram of each significant electrode by a multi-taper spectrum function (0.5 s moving window and 50 ms step size) for the first 5 s after motion onset and for the entire 30 s stimulation of each tactile stimulation block. To normalize the responses compared to baseline the obtained spectra of each trial were divided by the spectrogram of the baseline period, which was defined as the 2 s before each motion onset. Spectra were finally averaged across trials. Responses in time, starting 1 s before onset and lasting 5 s after offset stimulation, were averaged across the high frequency band (30–100 Hz) and the low frequency band (0–30 Hz). To estimate the time to peak (ttp) we computed for each electrode the first derivative of the z-score in the high frequency band within the first 5 s after motion onset.

The same type of analysis described for the tactile stimulation was applied to identify visually-responsive electrodes. For the visual functional localizer, visual active epochs were defined as the 0–0.5 s time period after stimulus onset for both the left and right hemifield stimulation, while baseline epochs were defined as 0.5 s before stimulus onset. To identify electrodes responding to the visual localizer, we compared, per electrode, the spectral power in the high frequency band for active visual epochs to high frequency band power during baseline epochs (paired t-test, p < 0.01 Bonferroni corrected for number of included electrodes).

Anatomical locations of the electrodes in subject-space were automatically extracted from the post-operative high- resolution CT scan via the ALICE software package (Branco et al. 2018). In brief, the CT scan was co-registered to the preoperative anatomical MRI scan (3D MPRAGE sequence, TR/TE 10 ms/4.6 ms; flip-angle 8°; FOV 240 × 240 × 160 mm; 200 slices, 0.8 mm isotropic voxel size), and electrodes were detected via the 3D-clustering algorithm of AFNI. Electrode coordinates where then projected to the cortical surface, to adjust for the brain shift that occurs as a consequence of brain surgery (Hermes et al. 2010), and rendered on the participant’s cortex. Electrode coordinates were also converted to Montreal Neurological Institute (MNI) space using AFNI (Fig. 2b). To define electrodes in visually-responding areas, we employed anatomical and functional criteria, which are described next.

### Anatomical Localization of Visual Cortex Electrodes

Visual maps of striate and extrastriate cortex of the participant were predicted from the preoperative anatomical MRI scan by the surface topology and an anatomically defined atlas of retinotopic organization (Benson et al. 2014; Benson and Winawer 2018). Using the alignment of the patient’s cortical surface to freesurfer’s fsaverage subject, atlas labels were interpolated onto the cortical surface via nearest-neighbour interpolation. Since the Benson atlas retains uncertainty outside of V1–V3 areas we predicted electrodes’ location using two additional freely available atlases: the (Wang et al. 2015) atlas, which contains probabilistic maps of visual topographic areas derived from retinotopic fMRI mapping; and the (Glasser et al. 2016) atlas, which is a whole-brain neuroanatomical parcellation based on functional, anatomical and diffusion MRI measurements from the Human Connectome Project (Fig. 5).

**Fig. 3:**
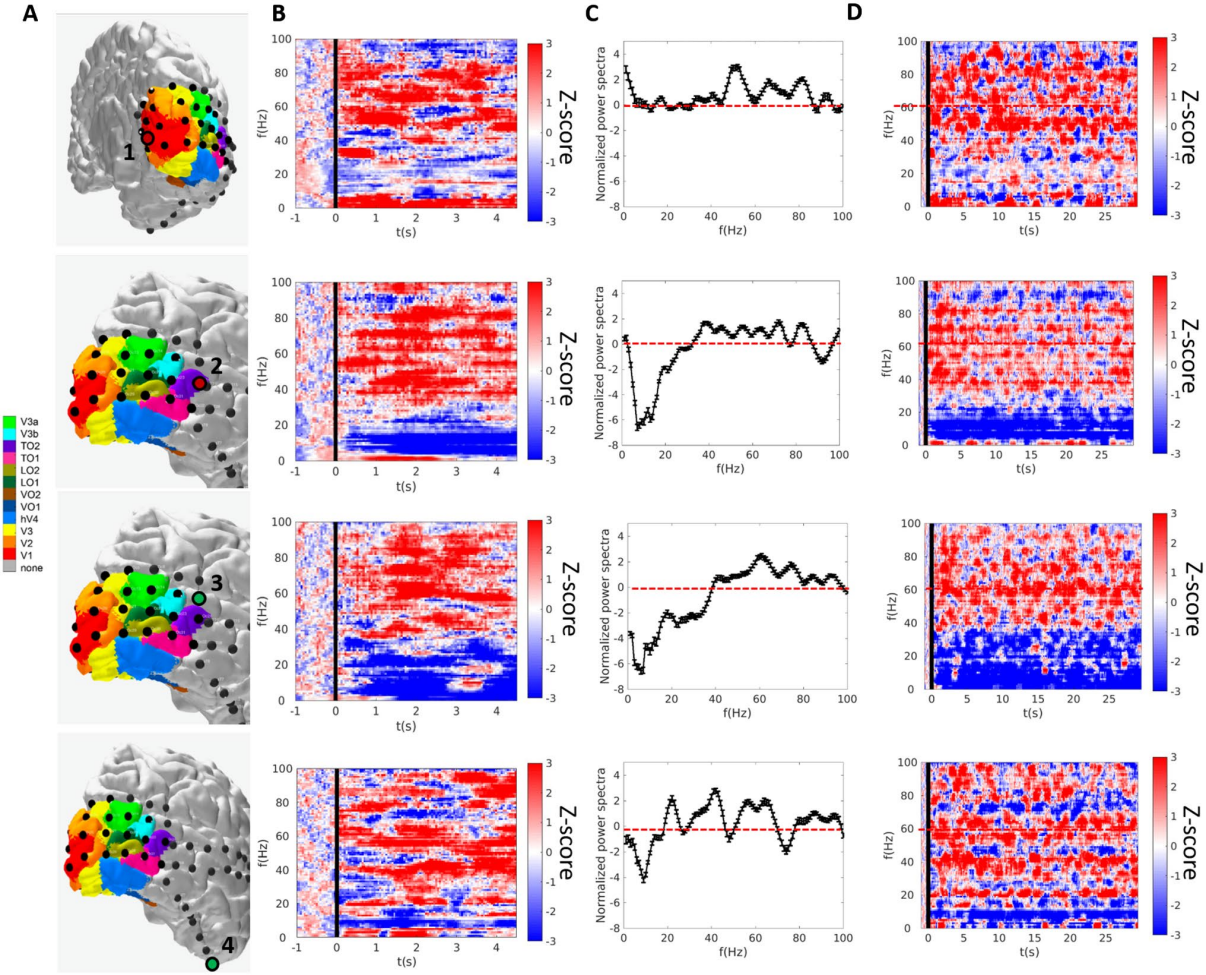
Power spectra in electrodes responding to tactile stimulation. **a** Significant electrodes rendered on the participant’s brain. (**b**) and (**d**) Spectrograms for tactile stimulation for 5 (**b**) and 30 (**d**) seconds of brushing of the right palm in the electrodes that showed a significant response to both visual and tactile stimulation (first and second rows, respectively) and for those electrodes responding only to tactile stimulation (third and fourth rows, respectively). The black line indicates the start of the brushing stimulation. Spectra are averaged across five trials, normalized by the 0–2 s baseline period before motion onset and cut off at a maximum of ± 3 log10 units. **c** Smoothed normalized power spectra and standard errors of the 0-5 s period after motion onset, averaged across the five motion trials in the 0–100 Hz frequency range

### Functional Localization of Visual Cortex Electrodes: pRF Mapping

Visual stimuli for the purpose of estimating the visual response properties of a population of neurons and to obtain retinotopic maps for individual electrodes (known as pRF mapping (Dumoulin and Wandell 2008; Kay et al. 2019)) were generated in Matlab and consisted of a bar stimulus covering 16.6° of visual angle. A grayscale contrast pattern was viewed through a bar aperture that swept across the visual field eight times in twenty-eight 0.85 s steps, which also included 8 blanks each. Each bar stimulus was displayed for 0.5 s followed by a 0.35 s blank period showing a grey mean luminance image. The participant completed two runs of the task during which she fixated on a cross located in the center of the screen and was instructed to press a button every time the fixation changed color (from green to red or red to green). Fixation cross color changes were created independently from the stimulus sequence and occurred at randomly chosen intervals ranging between 1 and 5 s. Stimuli were displayed on a 1920 × 1080 pixel NED MultiSync E221 LCD monitor positioned at 75 cm distance from the participant’s eyes. After common average referencing, the recorded ECoG data for each electrode were epoched into separate trials time-locked to the onset of each bar stimulus after which Welch periodograms were computed for the stimulus on period (0–0.5 s) using a 0.5 s window and 1 Hz sampling using Matlab R2018b. The obtained power density estimates were averaged across 30–200 Hz (avoiding the line noise frequency of 50 Hz and harmonics) using the geometric average in order to whiten the spectrum and to avoid bias towards the lower frequencies (Hermes et al. 2017), resulting in a single power estimate for each bar position in the stimulus sequence.

The pRF models were computed as described previously (Dumoulin and Wandell 2008), using the Compressive Spatial Summation (CSS) variant (Kay et al. 2019). Briefly, this involved (1) converting the stimulus into a sequence of binary contrast apertures, (2) projecting the contrast apertures onto the best-fitting 2D isotropic Gaussian pRF, and (3) passing the output through a static nonlinearity (power function) to predict the response. The pRF models were fit to each electrode’s ECoG responses separately by minimizing the difference between the predicted response and the observed response, using nonlinear optimization as implemented in the analyzePRF toolbox in Matlab (Kay et al. 2019). Before fitting the model, data were averaged across the two runs. Based on the resulting model fits, the following measures were calculated: (1) explained variance (R^2^), reflecting the goodness-of-fit of the predicted responses for bar stimuli passing through the Gaussian pRF and the measured ECoG responses; (2) pRF eccentricity, i.e. the distance of the center of the pRF from the center of the visual stimulus; and (3) pRF size, defined as σ/√*n*, whereby σ is the standard deviation of the pRF and *n* is the exponent of the power-law function. 95% confidence intervals on these estimates were obtained through a bootstrap procedure, whereby individual stimuli from the stimulus sequence were sampled with replacement and the fitting procedure was repeated for n = 100 bootstraps.

## Results

Visual stimulation to both the ipsilateral and contralateral hemifield elicited a significant increase in the high frequency band power range (30–100 Hz) in multiple ECoG electrodes (p < 0.01 Bonferroni corrected, Fig. 2a white dots). Two of these electrodes also exhibited a significant high-frequency band power (30–100 Hz) increase in response to tactile stimulation (Fig. 2, Electrodes 1 and 2, paired t-test, p < 0.01 Bonferroni corrected). These two electrodes were located in occipital cortices: one electrode was located on the medial surface of the occipital lobe near the calcarine sulcus (Electrode 1), and the other in lateral occipital–temporal cortex (Electrode 2). An additional two electrodes responded to tactile stimulation and were located on the right caudal part of the superior temporal area (Electrode 3, Fig. 2) and the anterior part of the ventral temporal cortex (Electrode 4, Fig. 2), respectively. These results show that tactile responses co-localize with visual responses in two electrodes that appear located in visual cortices. Indeed, according to MNI coordinates, these electrodes were located in secondary visual area V2 and the right middle temporal gyrus, respectively (see Fig. 2b).

To further establish the visual nature of cortices under these electrodes, we examined two additional sources of evidence. First, we compared the anatomical locations to a prediction of retinotopic maps derived from anatomy (Benson and Winawer 2018). According to this atlas, electrode 1 was located over the secondary visual cortex V2 and electrode 2 over the TO2 region (Electrodes 1 and 2, Fig. 2a), which is considered one of the retinotopic maps that are part of the hMT+ complex. For Electrode 2, we additionally compared the anatomical location to probabilistic maps of visual topographic areas (Wang et al. 2015) and a whole brain neuroanatomical parcellation (Glasser et al. 2016), which further demonstrated overlap of Electrode 2 with TO2 and parts of the MT + complex, respectively (Fig. 5).

Second, we estimated visual population Receptive Fields for all four electrodes responding to the tactile stimulation based on the independent pRF dataset (see Methods). Only electrodes 1 and 2 exhibited reliable pRF estimates as obtained from the fits of high frequency band power time courses (median R^2^ = 0.94, 95% confidence interval = [0.91, 0.96] and R^2^ = 0.22 CI = [0.14, 0.31], respectively; see Methods). The estimated pRF for electrode 1 was located precisely on the vertical meridian and—consistent with an early visual response profile—was relatively small and highly stable across bootstrapped model fits (median eccentricity 5.4° [5.3°, 5.6°], median pRF size 0.7° [0.6, 0.8°]). In comparison, the estimated pRF properties of electrode 2 (Fig. 2c) are consistent with a higher-level visual region, showing a larger, contralateral pRF that extended slightly into the ipsilateral field (median eccentricity 5.1° [1.4°, 16.6°], median pRF size 6.5° [1.1°, 15.6°]), consistent with TO2 pRF properties as reported by (Amano et al. 2009). Electrodes 3 and 4 did not have reliable pRF model fits, showing a median variance explained less than 20% and large variability in estimates across bootstrapped model fits.

**Fig. 4:**
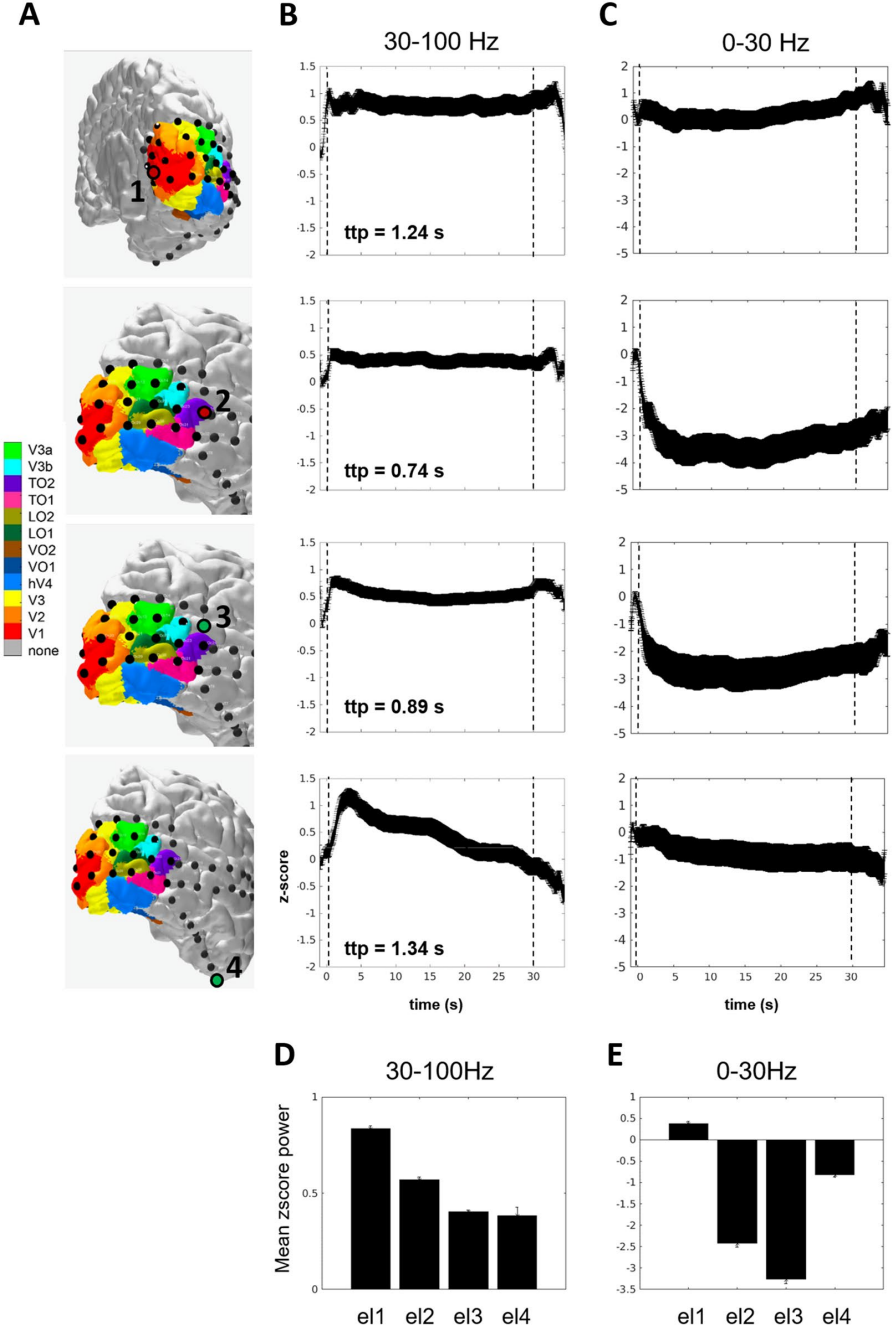
Mean z-score power in electrodes responding to tactile stimulation. **a** Significant electrodes rendered on the participant’s brain MRI. **b** Mean z-score power responses over trials in the high frequency band 30–100 Hz to 30 s tactile stimulation, starting at 0 s (dashed lines represent the stimulation onset and offset). **c** Mean z-score power responses over trials in the low frequency band 0-30Hz to 30 s tactile stimulation, starting at 0 s (dashed lines represent the stimulus onset and offset). **d** Mean z-score power responses and standard error of each selected electrode in the high-frequency band (30–100 Hz). Responses are averaged across the five trials of 30 s of tactile stimulation. **e** Same as D for the low-frequency band (0–30 Hz)

For the four electrodes significantly responding to tactile stimulation, we additionally characterized the z-scored spectral power change during the entire stimulation length (30 s) and for a 5 s window after tactile stimulus onset (Fig. 3). A broadband power increase in the 30–100 Hz frequency range was observed in all four electrodes (Figs. 3a–c and 4b) in response to tactile stimulation. In three out of the four electrodes, the spectral increase in the high-frequency band was accompanied by a decrease in the low-frequency range (Figs. 3b and 4c); a pattern which is thought to reflect a decline in neuronal inhibition concomitant with an increase in neuronal population spiking activity in both motor and visual cortices (see Fries et al. 2007 for review). The increase in high-frequency band power was sustained throughout the 30 s of tactile stimulation for electrodes 1 to 3 (Figs. 3d and 4) with an aftereffect sustained increase of few seconds after motion offset. The time-to-peak measure indicated a first peak in the lateral occipital–temporal cortex (Electrode 2, ttp = 0.74 s), then in the superior temporal sulcus (Electrode 3, ttp = 0.89 s), followed by secondary visual cortex V2 (Electrode 1, ttp = 1.24 s), and finally ventral cortex (Electrode 4, ttp = 1.34 s). Although time-to-peak measures should be taken cautiously, this pattern of observations is consistent with a network of responses starting earlier in the lateral occipital–temporal cortex and followed by responses in low-level cortices (i.e. secondary visual cortex, V2). Mean power change in the high and low frequency bands were consistent across the five blocks of 30 s of tactile stimulation as summarized in Fig. 4d, e.

**Fig. 5:**
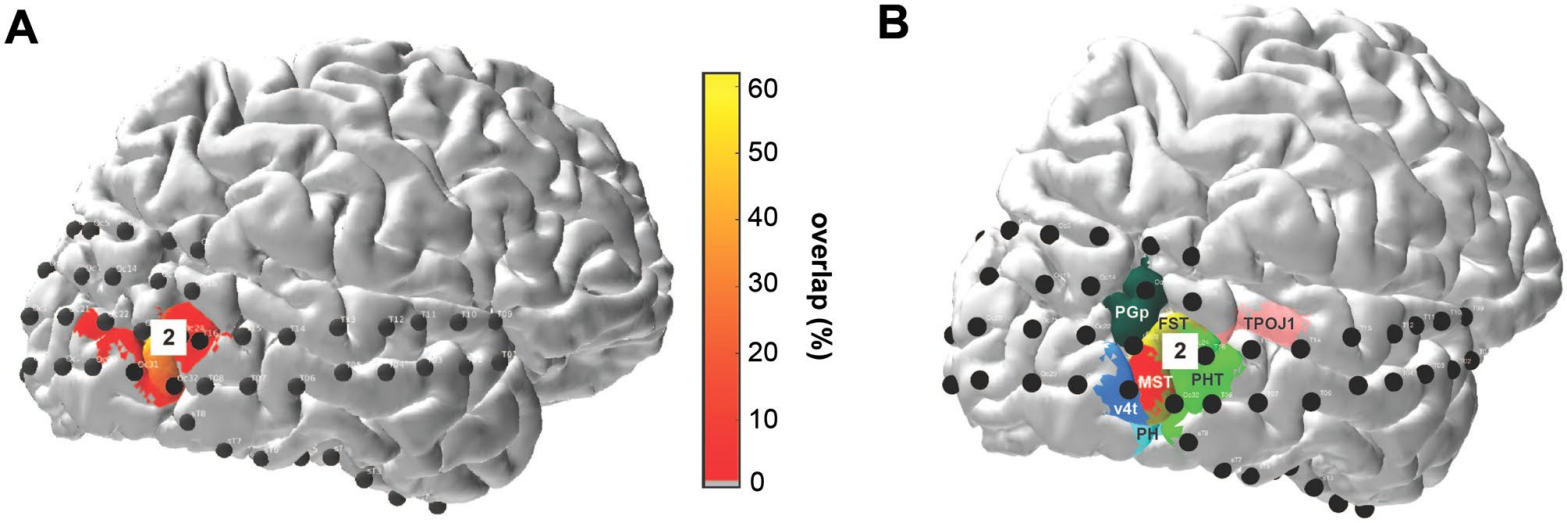
**a** Electrode location overlaid on a probability map of area TO2 derived from functional fMRI in 53 subjects (Wang et al. 2015). The color scale indicates the percentage overlap between subjects in the anatomical location of retinotopic map TO2. Note that the maximum overlap value is ~ 60%, indicating that there is no single vertex that is located in TO2 in 100% of the subjects measured by Wang et al. (2015). This is typical for higher-order visual regions whose precise locations vary across subjects in the normal population. **b** Electrode locations overlaid on a subset of brain regions in a neuroanatomical parcellation of the human brain derived from multi-modal MRI measurements in 210 healthy adults in the Human Connectome Project (Glasser et al. 2016). According to this parcellation, electrode 2 was located on the border of the fundus of the superior temporal area FST and putative human temporal area PHT. These areas were described by Kolster et al. (2010) as part of the retinotopic organization of the human middle temporal cortex

## Discussion

In the present case report, we documented tactile responses in two electrodes located in visually responsive areas using ECoG recordings in a human participant. These responses were evident as spectral power elevation in the high-frequency band range, which is considered as a proxy of spiking neuronal activity (Miller et al. 2009; Ray and Maunsell 2011). Such responses were observed in electrode sites located in low-level visual cortex and the anterior part of the lateral occipital–temporal cortex, as confirmed by both anatomical and functional localization approaches. Additional electrodes on the superior part of the middle temporal sulcus and the anterior ventral temporal lobe exhibited significant responsiveness to only tactile stimulation. Collectively, this pattern provides a level of support for considering specialized sensory cortices, such as visual cortex, task-sensitive. Indeed, these areas are recruited to process underlying process such as spatial, motion and self-processing independent of the sensory modality in which the stimuli are presented. This mechanism extends from higher-level cortices, such as the lateral occipital–temporal cortex, to low-level cortices, as secondary visual cortex V2.

In our study tactile stimulation elicited a significant high-frequency band increase in a specific electrode located in secondary visual area V2. Involvement of low-level visual cortex during tactile tasks has been demonstrated by various studies using fMRI and ECoG in both blind and sighted individuals (Sadato et al. 1996; Zangaladze et al. 1999; Ghazanfar and Schroeder 2006; Vetter et al. 2014). In addition, concurrent neuronal responses in visual cortex and primary somatosensory cortex S1 during tactile object discrimination has been reported in rats during whisker based tasks in the dark (Vasconcelos et al. 2011; Pereira et al. 2018) and in the macaque monkey (Guipponi et al. 2015). The specificity of the responses we observed in low-level visual cortex may reside on specific retinotopic-like maps in visual cortex for tactile location as recently shown for spatial sound (Norman and Thaler 2019). It has been also shown that the response fields of sensorimotor neurons are distributed over the entire hand (Goodman et al. 2019). Taken together with our observations, one could argue that this specific organization is reflected in visual cortex. Such a mechanism may suggest that sensory cortices are not entirely constrained to specific sensory modalities, but rather share the same functional architecture to respond to specific tasks that are sensory-independent in nature (Murray et al. 2016; Amedi et al. 2017).

In our direct neuronal recording in human visual cortex in response to tactile stimulation, we observed significant responses in the high-frequency band in the anterior part of the lateral occipital–temporal complex (Electrode 2, Fig. 2). The same electrode exhibited significant responses to visual stimulation, as shown by the visual functional localizer and pRF analysis performed (see Figs. 1 and 2). This result is consistent with several studies using both neurophysiological and neuroimaging techniques that emphasize the multisensory properties of the extrastriate cortex and the superior temporal sulcus STS. In particular, the hMT+ complex was shown to be recruited during both tactile (Blake et al. 2004; Beauchamp et al. 2007; Ricciardi et al. 2007; Ptito et al. 2009; Summers et al. 2009; Sani et al. 2010; Van Kemenade et al. 2014) and auditory motion stimulation (Poirier et al. 2005, 2006; Saenz et al. 2008; Collignon et al. 2011; Dormal et al. 2016; Kayser et al. 2017; Campus et al. 2017). In addition, involvement of the anterior part of hMT+ (MST/TO2 area) during tactile stimulation via median nerve stimulation has been recently reported by (Avanzini et al. 2016) using stereo-EEG recordings. By contrast, using fMRI, Jiang and colleagues (Jiang et al. 2015) reported weak or no activation during passive arm brushing in the hMT+ complex. Using single-subject analysis rather than group-level, they showed significant involvement of both the superior and the anterior parts of the entire complex in the superior temporal gyrus STS rather than the hMT complex. This difference highlights the possible confounds of performing group-level analysis in fMRI. The difference in localization among the STS and hMT+ may be also explained by the different types of stimulation used to study tactile motion responses. Indeed, arm brushing as used by (Jiang et al. 2015) may involve different pathways than hand palm stimulation employed in prior works (Beauchamp et al. 2007; Ricciardi et al. 2007) and the present study. The hand, similar to the eyes, plays a major role in sensory flow perception. Both tactile and optic information flow have a crucial role in object detection and on somatosensory processing of the self in space (Lacey and Sathian 2012, 2014; Kaliuzhna et al. 2016; Sathian 2016; Crollen et al. 2017; Harris et al. 2017), allowing one to navigate in the environment and to disambiguate self-motion from object motion. It has been shown that area MST/TO2 is involved in processing optic information flow (Duffy and Wurtz 1991; Kawano et al. 1994; Orban et al. 1995). Therefore, the involvement of this area for both visual and tactile stimulation may suggest a common shared neuronal substrate for both sensory modalities. In our study, we contend that the location of electrode 2 in the anterior part of the lateral occipital–temporal cortex is confined within the anterior part of hMT+ (see Figs. 2 and 5). Although time constraints prevented the patient from completing a separate localizer to functionally localize both hMT+ and STS, we did additionally localize the electrode anatomically using two different atlases, both of which were in agreement that the electrode was in the vicinity of hMT+ (see Fig. 5). According to one parcellation (Glasser et al. 2016), electrode 2 was located on the border of the fundus of the superior temporal area FST and the putative human temporal area PHT. Interestingly, the latter was recently shown to code for auditory motion and source location (Battal et al. 2019). Moreover, the electrode’s visual pRF properties are in line with the localization of the electrode within TO2. Both evaluations suggest the electrode localization in the anterior part of hMT+. However, more data are needed to confirm the anatomical localization based on atlases.

Significant responses in the high-frequency band to tactile stimulation were also measured in the anterior part of the STS (electrode 3, Fig. 2). This area has been extensively shown to activate during tactile motion stimulation (Ricciardi et al. 2007; Beauchamp et al. 2009; Jiang et al. 2015) and is considered related to object-centered and action-related motion-specific stimulation (Tanaka et al. 1993; Nelissen et al. 2006). The last site showing significant responses to tactile stimulation was unexpectedly observed in the anterior part of the ventral temporal cortex (electrode 4, Fig. 2). Among other functions, this area has been proposed to act as a single multisensory hub for verbal and non-verbal semantic processing (Ralph et al. 2016). We speculate that this area plays a role during tactile stimulation in order to process a semantic meaning of the action perceived by the participant.

### Limitations

Our results provide evidence that tactile stimulation elicits significant high-frequency band responses in (at least) two sites of visual cortices in the human brain. One limitation is that several control conditions would have been informative, but were impractical due to limited experimental time for performing ECoG studies in the patient. One could also argue that the significant responses we reported in visual cortex were related to mental imagery rather than tactile stimulation per se. However, because the tactile stimulation that were presented resemble motion processing due to the continuous brushing, if visual motion imagery was involved, a significant response to the task in the high frequency band in the well-known visual motion decoding area MT/TO1 would have been expected. Indeed, among others we recently showed using ECoG measurements in other patients that the loci of visual motion processing are located more posteriorly in MT/TO1 (Gaglianese et al. 2017a, b).

Our findings are limited to a single patient. However, ECoG single subject cases have been determined to be informative before (Harvey et al. 2013; Van der Stigchel et al. 2019) in recognition of the high sensitivity of the ECoG recordings and the uniqueness of the information ECoG data provide on human neuronal population responses. The implantation of subdural electrodes allows to directly measure task-related neural activity in humans, enabling us to provide valuable information on brain processing and function. Future studies using well-controlled and unified tactile and visual stimulation, combining both neurophysiological and imaging results will be essential to disentangle the sensory specificity of motion-sensitive areas in the human brain.

## Conclusions

This study provides evidence of a direct neuronal response to tactile stimulation in brain areas canonically thought to be devoted to vision. This finding, particularly in a normally-sighted individual, is of great interest for elucidating whether the functional recruitment and specialization of the visual system is independent of the sensory modality in which the stimuli are delivered. The fact that brain areas, and in particular the neuronal populations within the same area, respond to a specific type of information independently of the modality conveying the sensory input, may provide knowledge that is essential for our understanding of the neuronal mechanisms underlying sensory processing in the human brain and can have a crucial impact on novel research on sensory substitution devices for rehabilitation in sensory-impaired individuals.
